# Application of Near-Infrared Spectroscopy and Hyperspectral Imaging Combined with Machine Learning Algorithms for Quality Inspection of Grape: A Review

**DOI:** 10.3390/foods12010132

**Published:** 2022-12-27

**Authors:** Weixin Ye, Wei Xu, Tianying Yan, Jingkun Yan, Pan Gao, Chu Zhang

**Affiliations:** 1 College of Information Science and Technology, Shihezi University, Shihezi 832003, China; 2 College of Agriculture, Shihezi University, Shihezi 832003, China; 3 School of Information Engineering, Huzhou University, Huzhou 313000, China

**Keywords:** near-infrared spectroscopy, hyperspectral image, grape quality, machine learning

## Abstract

Grape is a fruit rich in various vitamins, and grape quality is increasingly highly concerned with by consumers. Traditional quality inspection methods are time-consuming, laborious and destructive. Near-infrared spectroscopy (NIRS) and hyperspectral imaging (HSI) are rapid, non-destructive and accurate techniques for quality inspection and safety assessment of agricultural products, which have great potential in recent years. The review summarized the applications and achievements of NIRS and HSI for the quality inspection of grapes for the last ten years. The review introduces basic principles, signal mode, data acquisition, analysis and processing of NIRS and HSI data. Qualitative and quantitative analysis were involved and compared, respectively, based on spectral features, image features and fusion data. The advantages, disadvantages and development trends of NIRS and HSI techniques in grape quality and safety inspection are summarized and discussed. The successful application of NIRS and HSI in grape quality inspection shows that many fruit inspection tasks could be assisted with NIRS and HSI.

## 1. Introduction

The grape (*Vitis vinifera* L.) is a woody vine of the Vitis genus in the family of Vitaceae. They are cultivated worldwide, and most are concentrated in the northern hemisphere [1]. Grape is a kind of world economic crop fruit, and it is one of the essential components of the agricultural economy for immense dietary purposes [2]. It is popular due to its rich vitamins and unique flavor. Grapes are consumed as fresh fruit (table grapes), dried fruit (raisins), juice, and a large proportion of grapes are used to make wine [3]. Especially grape quality and variety are the main determinants of wine quality, and pigments extracted from the grape skin are reused as functional food ingredients in other food products [4]. Besides, it is adaptable, widely distributed, and easy to manage, with high yield, rich nutrition, multiple uses and high economic benefits in the local and national economy. Increasingly, consumers are concerned about quality, and it has a significant influence on grape winemaking [5]. Therefore, grape quality detection is vital, and it is helpful for fully utilizing grapes of all grades. Generally, good quality grapes are used for food, while poor quality grapes are used as fertilizer or fuel, which also responds to environmental rules and contributes to sustainable economic development [6].

Grape quality means the internal factors, such as sugar content (roughly equivalent to soluble solids content, SSC), acidity and phenolic, and external factors, such as color, firmness (Durofel index, DI) and surface defect. Grape safety refers to the pesticide residue and decay on the surface here. Generally, the method of fruit quality detection has been divided into conventional (destructive) techniques and advanced and green (non-destructive) techniques. Currently, the main conventional techniques include various chemical inspection methods, such as High-Performance Liquid Chromatography (HPLC) [7], Gas Chromatography (GC) [8], Mass Spectrometry (MS) [9], and Gas Chromatography-Mass Spectrometer (GC-MS) [10], etc. They are diagnostic analytical methods at the molecular level, and are used combined with NIRS, HSI and other scientific studies. Besides, they are time-consuming, laborious, cost and required the complex operation and process, and they are unsuitable for the extensive detection of fruits in the real world. Thus, the development of the fruit economy and application is seriously restricted.

Researchers have been exploring green, rapid and accurate non-destructive techniques (NDTs) to monitor agro-product quality in the past decades. Common non-destructive techniques are mainly imaging-based, spectroscopy-based, and other non-destructive methods [6]. Among them, imaging-based approaches include RGB imaging, multispectral imaging, hyperspectral imaging, backscattering imaging, thermal imaging (TI), fluorescence imaging, magnetic resonance imaging, X-ray and Ultrasonic imaging. Spectroscopy-based techniques contain NIR spectroscopy and Raman spectroscopy. Other non-destructive methods are the electronic nose, electronic tongue, dielectric, and acoustic [6]. Non-destructive detection of quality would allow the fruit industry to provide better tasting grape fruit to consumers, which would improve competitiveness and profitability in the market.

**Figure 1:**
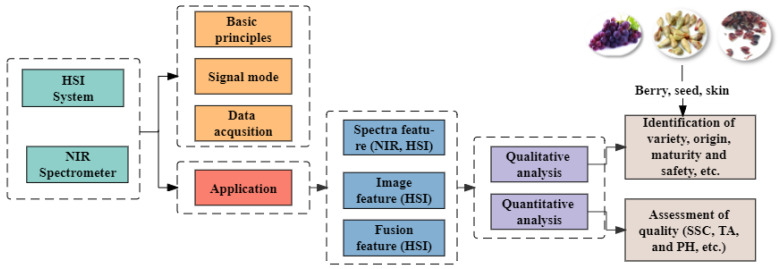
The mainframe of the review.

In recent years, near-infrared (NIRS) and hyperspectral imaging (HSI) have been widely used in agriculture [11], the food industry [12] and the medical industry [13], etc. However, comprehensive literature on grape quality inspection of NIRS and HSI techniques has not been available to our knowledge. The review was conducted by reading and analyzing the literature of the last ten years to comprehensively understand the quality inspection for grapes using NIRS and HSI techniques. The basic principles, data processing methods, calibration model and research results are summarized and analyzed. The development trends and shortcomings of the NIRS and HSI techniques in fruit quality inspection are discussed. Based on the NIRS and HSI, this is the first time that a comparative study between entire grapes, skins and seeds has been evaluated with this purpose. The frame of the review is shown in Figure 1.

## 2. Introduction to NIRS and HSI Technologies

### 2.1. Basic Principles and Signal Mode

Near-infrared spectroscopy (NIRS) is vibrational spectroscopy, and it covers the wavelength range of 780–2500 nm (12500–4000 cm^−1^), which presents the molecular bond vibrations, including hydrogen-containing O–H, C–H, N–H and C-O groups [14,15]. It is a commonly used technique for non-destructive quality inspection of agro-products. When radiation hits a sample, the incident radiation may be reflected, absorbed and transmitted. The relative contribution of each phenomenon depended on the sample’s chemical constitution and physical parameters [16].

Hyperspectral imaging (HSI) is another commonly used advanced technique for the non-destructive quality inspection of agro-products, which combines spectroscopy and the conventional imaging technique. It can obtain three-dimensional (3D) data information from the sample simultaneously, including the one-dimensional (1D) spectral information and two-dimensional (2D) spatial (image) information, achieving the unity of the map and spectrum [17,18], which is helpful to obtain qualitative and quantitative information about the product.

In signal mode, the interaction between light and biological tissues proves to be highly complicated [19]. When the incident light hits the grape, the part of light will be reflected on the surface directly, the remaining light penetrates the tissue interior, where they are absorbed and scattered. Before the light is absorbed, light spreads out in all directions, forming transmission or diffuse reflection. Among them, light exiting from the same side (the angle between the incident light and the emergency light is less than 90°) is defined as diffuse reflection, and the light exiting from the other side (the angle between the incident light and the emergency light is equal to or greater than 90°) is called transmittance [20].

**Figure 2:**
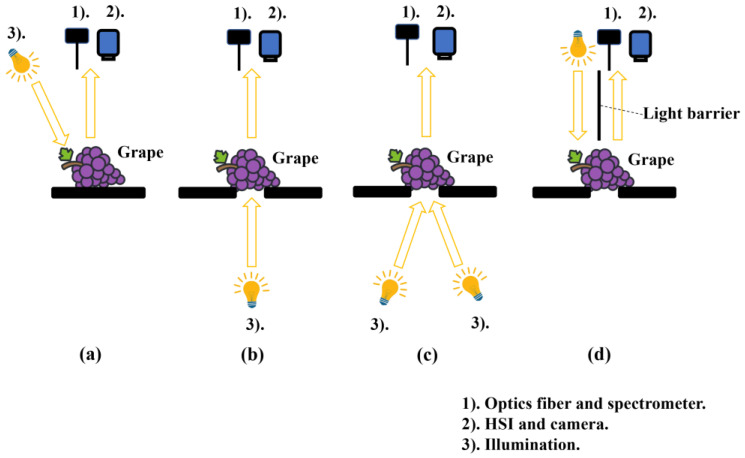
Detection mode of obtaining data. (**a**) Diffuse reflectance. (**b**) Full transmittance. (**c**) Partial transmittance. (**d**) Interactance.

Generally, NIRS and HSI present the three detection modes, including reflectance, transmittance, and interactance [21,22], as shown in Figure 2. Generally, reflectance refers to diffuse reflectance, and illumination and the detector are on the same side, as is shown in Figure 2a. The full transmission mode is usually configured with the illumination and detector on opposite sides of the sample, and the illumination, detector and sample are in one line, as is shown in Figure 2b. However, regarding partial transmittance, the illumination, detector and sample are not in a line, as is shown in Figure 2c. In interactance mode, the illumination and detector are installed on the same side of the sample and parallel to each other. In addition, a light barrier is placed between the illumination and the detector to prevent interference, as is shown in Figure 2d.

### 2.2. Data Acquisition

Near-infrared spectroscopy (NIRS) data is acquired by a near-infrared (NIR) spectrometer. Generally, it consists of four modules: A light source, detector, sample stage, and light-isolating [23]. Based on the application, NIR spectrometers can be divided into laboratory, portable, and online spectrometers [14]. In recent years, portable vis/NIR systems have been developed due to innovations in optical system design and miniaturization for their friendly use directly in the field [24]. Specific online applications include laboratory scale, semi-industrial pilot scale applications, and industrial conditions [19,25].

Hyperspectral imaging (HSI) data is obtained by a HSI system. A typical HSI system generally contains four modules: An imaging unit, an illumination source, a carrier stage, and a computer with corresponding control software [26]. The imaging unit consists of the charged-couple device (CCD) camera. The illumination unit is composed of a tungsten-halogen lamp to provide illumination. A sample was presented in the sample stage, and the data were acquired and processed in the computer equipped with relevant analysis software. Three ways to obtain hyperspectral images are developed, including point scan mode, line scan mode and area scan mode. To some extent, the NIR spectrometer and hyperspectral image system have their advantages, disadvantages and application fields, and the main similarities and differences are shown in Table 1.

### 2.3. Data Analysis and Processing

**Figure 3:**
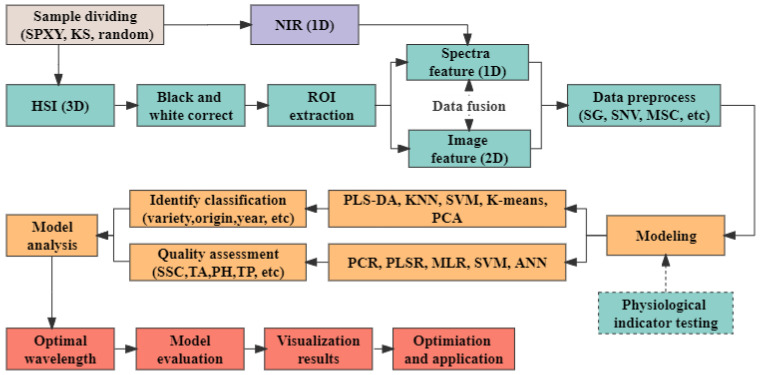
Data processing process of NIRS and HSI.

Data analysis includes qualitative and quantitative analysis. The qualitative analysis of the sample provides non-quantifiable attributes, while quantitative analysis can extract quantitative sample information from the NIRS and HSI data. Generally, the analysis processing of NIRS and HSI data is shown in Figure 3. The main steps of data processing are summarized as follows:

(1) Division of sample set. When the sample set is divided, the sample’s content distribution, gradient, and physical and chemical properties should be considered to improve the calibration model’s stability and expand the model’s practical application. The main dividing methods include Kolmogorov-Smirnov (KS) [27], sample set partitioning based on joint x-y distance (SPXY) [28] and random ratio.
(2) Collection and extraction of data. The NIRS and HSI data are obtained, and the chemical analysis values are measured. NIRS acquires spectral data and directly processes it later. Regarding HSI data, it will be corrected with a black and white reference image to eliminate random noise signals caused by a light source or power supply [29]. The region of interest (ROI) is extracted using masking to remove the background.
(3) Data preprocessing. The spectral signal obtained by the detector includes various non-target factors, such as high-frequency random noise, baseline drift, stray light, etc. Therefore, the obtained spectra should be reasonably pretreated before data analysis for the specific spectral measurement and sample. Normalization, Savitzky-Golay (SG) [30], Standard Normal Variate (SNV) [31], and Multiplicative Scatter Correction (MSC) [32] have been used widely to reduce noise. Normalization is to map the data to the range to unify the dimension and speed up the calculation. Besides, it could reduce the spectral difference caused by the varying height of the sample surface. SG can eliminate spectral noise, such as baseline offset, tilt, reverse, etc. SNV is commonly used to attenuate the slope variation of spectra. MSC is applied to remove the undesirable scatter effect. Besides, derivative processing, Fourier Transform (FT), Wavelet Transform (WT), etc., are applied in some cases.
(4) Establishment of calibration models. For the qualitative analysis, the calibrations are conducted by the classification model using the sample label (variety, origin, year, etc.) as the dependent (Y) variable and grape spectra as the independent (X) variable [33]. Classification calibrations models are built, such as Partial least squares discriminant analysis (PLS-DA) [34], K-nearest Neighbor (KNN) [35], Support Vector Machine (SVM) [36], K-means [37], Artificial neural networks (ANN) [38], etc. For quantitative analysis, calibrations were developed by the regression model using the fruit physicochemical attribute as the dependent (Y) variable and grape spectra as the independent (X) variable [33]. Regression calibrations models are established, such as Partial Least-square Regression (PLSR) [39], Multiple Linear Regression (MLR) [40], SVM [36], ANN [38], Principle component regression (PCR) [41], etc. For NIRS, the input data is the principal component of the grape spectra. Regarding HSI, it is spectra, images, or a combination of spectra and image features.
(5) Evaluation of the calibration model. The model conducted is evaluated for its reliability and generalization capability with external validation data sets or/and cross-validation techniques. There are some evaluation indices: Accuracy (acc), precision, recall, and F-score, etc., for qualitative analysis; the correlation coefficient (R), coefficient of determination (R^2^/RSQ), root mean squared error (RMSE), residual predictive deviation (RPD), etc., for quantitative analysis.
(6) Prediction of unknown samples [42]. The unknown samples were scanned to obtain NIRS and HSI data, and their contents were calculated by models established and evaluated.

## 3. Applications

In recent years, NIRS and HSI technologies have been widely used to assess agro-product quality, enabling a better understanding of the sample’s composition, physicochemical characteristics, and internal structure. In this section, qualitative research of NIRS and HSI in grapes were analyzed, mainly including discrimination of the variety, vintage, vineyard, maturity, quality grading, status evaluation, seeded or seedless, safety inspection, etc., which are summarized in Table 2, Table 3 and Table 4. Quantitative analysis in grape berries, grape seed, grape skin and grape pomace mainly consists of internal quality assessment, sensory and preference analysis of consumers, biochemical component detection, etc., summarized in Table 5 and Table 6. Internal quality parameters discussed here consist of texture and flavor, which are significant for determining the nutritional values and marketability of agro-products. The relationship between spectra data and the sensory preference analysis of consumers will be explored. Biochemical composition detection consists of evaluating extractable polyphenols (total phenolic, anthocyanins and flavanols), amino acids and glycosylated aroma compounds. Besides, NIRS obtains the spectra data, and HSI acquires the spectra and image data. Thus, relevant research was summarized and compared in the section based on the spectra feature, image feature (color space, texture and morphology) and fusion feature.

### 3.1. Spectral Feature Analysis

#### 3.1.1. Qualitative Analysis

##### Variety Identification

Grape cultivars, especially wine grapes, play an essential role in winemaking, directly affecting grape wine quality. Cheng et al. used HSI to identify varieties of six white and six red wine grapes during the ripening period. The mahalanobis distance was employed to remove outliers of the spectral data. Six different pre-treatments (MSC, S-G filter, 0–1 normalization, SNV, first derivative and second derivative) were applied. Principal Component Analysis (PCA) loading was used to select effective wavelength. SVM, Random Forest (RF), and AdaBoost models were built with effective and full wavelength as prediction models. The S-G Filter + PCA + SVM achieves the best classification result, with the accuracy of 81.09% and 90.01% for white and red grapes [43]. Xu et al. used HSI based on ensemble empirical mode decomposition discrete wavelet transform (EEMD-DWT) to identify grape varieties. EEMD-DWT, SG, DWT and EMD were applied to denoise, and Competitive Adaptive Reweighted Sampling (CARS) and Successive Projections Algorithm (SPA) was involved in extracting the feature wavelength. Finally, SVM was built to identify the varieties of grapes, with the best accuracy of 99.3125% based on a model of EEMD-DWT-CARS-SPA in Monte Carlo (MC) experiments [44]. Besides, the chemical composition of seeds changes during grape ripening, affecting the wine’s sensory properties [45]. Zhao et al. used near-infrared HSI with multivariate analysis to non-destructively and rapidly discriminate and visualize different grape seeds. The effective wavelengths were extracted by the PCA loadings of the first six PCs. SVM was applied to establish classification, with a calibration accuracy of 94.3% and a prediction accuracy of 88.7% [46]. Rodríguez-Pulido et al. used NIR hyperspectral imaging combined with multivariate analysis methods PCA and General Discriminant Analysis (GDA) to predict the variety of grape seeds. The study shows a good result, with the accuracy of 100% using full spectra and 96% using the six selected wavelengths [47]. Quijada-Morín et al. successfully divided grape seeds into seven groups through hyperspectral imaging characteristics with k-means [48].

**Table 2:** Qualitative analysis of near-infrared spectroscopy (NIRS) in grape.

Variety	No	Mod	S/I	Attribute	Ext	Object	Model	Application	Best Result (Accuracy%)	Reference
‘Kyoho’	86	inter	S	seed or seedless	No	berry	PLS-DA	identify seed or seedless	acc = 93.10%	[49]
Graciano (two origins)	84	refl	S	phenolic in skin and seed	No	seed, skin, berry	DPLS	identify the origin	acc = 95%, 66%, 93% (DPLS, seed, berry, skin)	[50]
Manicure Finger, Ugni Blanc	341	inter	S	SSC, TP, CIELAB	No	cluster	PLS-DA	quality grade	77.00–94.00%	[51]
Tempranillo, Syrah (two years)	400	drefl	S	TP, anth, flav	No	berry, skin	LDA, DPLS, Pearson	quality assessment	acc = 87.0, 91.3, 91.3 (LDA), others are poor result	[52]
Syrah, Cabernet Sauvignon	1008	refl	S	TSS, yellow flavonoids, anth	No	berry	PCA-LDA, PCA-QDA, LDA_Mahalanobis, PLS-DA	maturity evaluation	acc = 93.15% (PLS-DA), 92.86% (LDA), 92.26% (QDA), 92.26% (LDA_Mahalanobis)	[53]
Manicure Finger Ugni Blanc	540	drefl	S	L*a*b, SSC, TP	SPA, CARS	berry	PCA SVM-DA	maturity evaluation	acc = 90.00% (MF) acc = 100.00% (UB) (SSC-CARS-SVM-DA)	[54]
Sangiovese	400	absorb	S	SSC, TA, DI anth	No	berry	PCA	maturity evaluation	clear clusters (PC1 for 93.42%, PC2 for 4.72%)	[55]
Pedro Ximénez, Cabernet Sauvignon	24	refl	S	SSC, PH, TA, MA, reducing-sugar, tartaric acid	No	bunch	PLS-DA	maturity evaluation	acc = 79.00–100.00%	[56]

Variety refers to grape varieties. No means the number of the sample in the research. Object means the object-wise: single berry, a bunch, grape skin and grape seed. S/I means spectra and image features, respectively. Mod means the signal mode: transmittance mode (trans), interactance mode (inter), reflectance mode (refl), diffuse reflectance (drefl), and diffuse transmittance (dtrans). Ext means the approach to extracting effective wavelength. Best result means the best perform of model in the research. Application means the main relevant research contents in this cited literature. Reference means the reference resources.

**Table 3:** Qualitative analysis of hyperspectral imaging (HSI) in grape.

Variety	No	Mod	S/I	Attribute	Ext	Object	Model	Application	Best Result (Accuracy%)	Reference
Garnacha (two vineyards), Graciano, Mazuelo, Tempranillo	50	refl	SI	Chromatographic, color, NIR, fusion data	No	berry	Stepwise-LDA	identify grape variety	acc = 88%, 54%, 100%, 100% (internal validation) acc = 86%, 52%, 86%, 86% (external validation)	[57]
Six white and red wine grapes	5640	refl	S	No	PCA	berry	AdaBoost, SVM, RF	identify grape variety	acc = 81–93.00%	[43]
Hutai, Kyoho, Muscat, Summer black	480(120 * 4 varieties)	refl	S	No	CARS, CARS-SPA, MCCV	berry	SVM	identity grape variety	acc = 99.3125% (CARS-SPA)	[44]
Tempranillo, Syrah, Zalem-a (two soils)	56	refl	S	No	PCA	seed	GDA	identity grape seed variety	acc = 100% (full wavelength), ≥96% (selected wavelength)	[47]
Hongtizi, Meirenzhi, Jufeng	500	refl	S	No	PCA	seed	SVM	identity grape seed variety	acc = 88.70%	[46]
Tempranillo	1232	refl	S	Flavanolic	PCA	seed	k-means	predict flavanolic	k-means clustering great	[48]

Variety refers to grape varieties. No means the number of the sample in the research. Object means the object-wise: single berry, a bunch, grape skin and grape seed. S/I means spectra and image features, respectively. Mod means the signal mode: transmittance mode (trans), interactance mode (inter), reflectance mode (refl), diffuse reflectance (drefl), and diffuse transmittance (dtrans). Ext means the approach to extracting effective wavelength to build mode. Best result means the best performing model in the research. Application means the main relevant research contents in this cited literature. Reference means the reference resources.

**Table 4:** Safety inspection of NIRS and HSI in grape.

Variety	No	Mod	S/I	Attribute	Ext	Object	Model	Application	Best Result (Accuracy%)	Reference
Chardonnay, Grillo, Inzolia, Viognier, Nero’d’Avola, Syrah	1235 healthy, 1324 diseased	refl	S	Healthy and diseased status	No	bunch	PLS-DA	phytosanitary status evaluation	acc = 89.80–94.00%	[58]
table grape	686	refl	S	no, single and double dose of pesticide	PCA, LASSO, Elastic Net regularization	cluster	ANN, SVM, RF, XGBoost	identity pesticide level	acc = 91.98% (SVM-LASSO)	[59]
cabernet sauvignon, Red grape, Munage	1071	refl	S	four mixed pesticide levels	No	cluster	RF, LR, SVM, ResNet	identity pesticide level	acc > 93.00%	[60]

Variety refers to grape varieties. No means the number of the sample in the research. Object means the object-wise: Single berry, a bunch, grape skin and grape seed. S/I means spectra and image features, respectively. Mod means the signal mode: Transmittance mode (trans), interactance mode (inter), reflectance mode (refl), diffuse reflectance (drefl), and diffuse transmittance (dtrans). Ext means the approach to extracting effective wavelength to build mode. Best result means the best performing model in the research. Application means the main relevant research contents in this cited literature. Reference means the reference resources.

**Table 5:** Quantitative analysis of near-infrared spectroscopy (NIRS) in grape.

Variety	No	Mod	Attribute	Ext	Object	Model	Application	Best Result(R^2^)	Reference
Grape mash (36 varieties)	168	refl	Fructose, PH Glucose, TA, Glycerol, MA, Gluconic acid, Ergosterol, Ethanol, acetic acid, Tartaric acid, Laccase activity	No	berry	PLSR	predict grape mashes composition	R^2^ = 0.873 (Relative density), 0.836 (Glycerol), 0.851 (Ergosterol), 0.345 (TA), PH (0.393)	[25]
Tannat (3 years)	56	refl	glycosylated aroma compounds	No	homogenized, juice	PLSR	predict glycosylated aroma compounds	RPD > 1.5 (5 and 4 norisoprenoids compounds, in homogenized and juice)	[61]
Cabernet Sauvignon, Syrah	1008	refl	TSS, anth, yellow flavonoids	No	berry	PCR, MLR, PLSR	quality evaluation	≥0.90 (TSS and anthocyanins); ≥0.70 (flavonoids)	[53]
Autumn royal, Timpson, Sweet scarlett	450	refl	Dry matter (DM), TSS/SSC	No	berry	PLSR	quality evaluation	R^2^ = 0.83,0.81 (DM), 0.97, 0.95 (TSS) for two spectrometers	[62]
Jufeng	115	dtran	SSC	No	bunch	PLSR	quality evaluation	R = 0.83	[63]
Tempranillo (laboratory, field)	1643	refl	TSS	No	berry	PLSR	quality evaluation	RMSEP = 1.42°Brix, SEP = 1.40°Brix (laboratory);1.68°Brix, 1.67 Brix (field)	[64]
Sangiovese	9600	drefl	Brix, Babo, TS, glucose, fructose, density, TA, tartaric acid, pH, MA, anth, TP, gluconic acid, assumable nitrogenm	No	berry	PLSR	quality evaluation	R^2^ = 0.93 (°Brix), 0.93 (°Babo), 0.94 (TS), 0.93 (glucose), 0.55 (TA), 0.92 (fructose), 0.91 (density), 0.66 (PH), 0.76 (anth)	[65]
Tempranillo	144	refl	TSS, anth, total polyphenols	PCA	bunch	PLSR	predict TSS, anth, total polyphenols	R^2^ = 0.95, 0.79, 0.43	[66]
Grenache	128	refl	TSS, amino acid	No	cluster	PLSR	predict amino acids and TSS	R^2^~0.60 (asparagine, tyrosine proline in 570–1000; lysine, tyrosine, proline in 1100–2100), 0.90 (TSS)	[67]
Ruby Seedless grape	700	refl	SSC	No	berry	PLSR, LS-SVM	predict SSC	R^2^ = 0.889~0.918 (LS-SVM); 0.874~0.907 (P-LSR)	[68]
Syrah, Tempranillo	400	drefl	TP, anth, flava	No	berry, skin	MPLSR	quality evaluation	poor results	[52]
table grape cv Italia	682	drefl	SSC	No	berry	PLSR	sensory analysis	R^2^ = 0.85 (cross-validation); 0.82 (external validation)	[69]
Autumn Royal, Victoria	350	refl	TSS/SSC, TA	No	berry	PLSR	predict consumer preference driving factors	R^2^ = 0.5732 (TA), 0.8304 (TSS)	[70]
Thompson seedless, Regal seedless, Prime seedless	338	drefl	TSS, TA, PH, TSS/TA, BrimA	No	bunch	PLSR	predict maturity and sensory parameters	R^2^ = 0.71, 0.33, 0.57, 0.28, 0.77	[71]
Graciano red grape (two vineyards)	150	refl	taste, texture, visual, olfactory feature	No	seed skin	MPLSR	predict sensory parameters and harvest time	seed (4.5% for hardness, 8.7% for colour), skin (9.8% for tannic intensity, 13.7% for astringency	[72]
Corvina	300	refl	TSS, DI, weight loss	No	berry	PLSR, PCA	predict withering quality	R^2^ = 0.62, RPD =1.87 (TSS); 0.56, 1.79 (firmness)	[73]
Manicure Finger (MF), Ugni Blanc (UB)	540	drefl	L*a*b, SSC TP	SPA CARS	berry	PLSR, LS-SVM	quality evaluation	R^2^ = 0.531~0.929 (LS-SVM), 0.520~0.897 (PLS); 0.897, 0.929 ( SSC, UB)	[54]
Sangiovese	400	absorb	SSC, TA, DI, anth	No	berry	Pearson	quality evaluation	R^2^ = 0.92 (SSC), 0.87 (TA), 0.89 (DI), 0.68~0.97 (anth)	[55]
‘Kyoho’ grape	172	inter	DI, SSC, PH,	No	berry	PLSR	quality evaluation	R^2^ = 0.7427, 0.7804 (DI); 0.6276, 0.7676 (PH); 0.6926, 0.8052 (SSC)	[49]
Manicure Finger, Ugni Blanc	341	inter	SSC, TP, LAB	No	berry	PLSR	quality evaluation	R^2^ = 0.735, 0.823 (SSC, TP)	[51]

Variety refers to grape varieties. No means the number of the sample in the research. Object means the object-wise: single berry, a bunch, grape skin and grape seed. S/I means spectra and image features, respectively. Mod means the signal mode: transmittance mode (trans), interactance mode (inter), reflectance mode (refl), diffuse reflectance (drefl), and diffuse transmittance (dtrans). Ext means the approach to extracting effective wavelength to build mode. Best result means the best performing model in the research. Application means the main relevant research contents in this cited literature. Reference means the reference resources.

**Table 6:** Quantitative analysis of hyperspectral imaging (HSI) in grape.

Variety	No	Mod	S/I	Attribute	Ext	Object	Model	Application	Best Result (R^2^)	Reference
Zalema, Te-mpranillo	95	refl	S	flav	No	seed	PLSR	predict flavanols in grape seeds	R^2^ = 0.88 (1 variety); 0.85 (2 varieties)	[45]
Syrah, Tempranillo	99	refl	S	anth	No	berry	MPLSR	Screen anthocyanins	R^2^ = 0.86	[33]
Cabernet Sauvignon	46	refl	S	anth	PCA	skin	PLSR	detect anthocyanin concentration	R^2^ = 0.65	[74]
Cabernet Sauvignon	120	refl	S	anth	PLSR	berry	PLSR SVR	predict the anthocyanin content	R^2^ = 0.94 (SVR)	[75]
Touriga Franca, Tin-ta Barroca, Touriga Nacional	552	refl	S	anth, PH sugar	PCA	bunch	SVR	prediction of oenological parameters for different vintages and varieties	R^2^ = 0.89 (anth);0.81 (PH); 0.90 (sugar)	[76]
Syrah, Tempranillo	200	refl	S	TP, anth, flav	PCA	skin	MPLSR	screen of extractable polyphenols in red grape skins	R^2^ = 0.82 (TP), 0.79 (anth); 0.82 (flavanol),	[77]
Tempranillo	144	refl	S	SSC/TSS, anth	PCA	berry	SVM	Evaluate TSS and anthocyanin concentration	R^2^ = 0.92 (TSS); 0.83 (anth)	[78]
Sangiovese	429	refl	S	SSC	VIP	berry	PLSR, PLS-DA	Evaluate SSC and assess harvest time	R^2^ = 0.77 (PLSR) acc = 0.86–91% (PLS-DA)	[79]
Kyoho grapes	240	refl	S	SSC/TSS	CARS, IRIV, V-MDRC	berry	LSSVM, PLSR	detect TSS	R2 P = 0.93 (VMD-RC-LSSVM)	[80]
Sangiovese	33	drefl	S	SSC	No	berry	PLSR	predict SSC in the field	R^2^ = 0.75, RMSECV = 0.84	[81]
Tempranillo	144	refl	S	TSS, TA, PH, anth, MA, total polyphenols, ftartaric acid	No	cluster	PLSR	predict internal parameters	R^2^ = 0.82 (TSS), 0.81 (TA), 0.61(PH), 0.62 (Tartaric acid), 0.84 (MA), 0.88 (anth), 0.55 (Total polyphenols)	[82]
Sugarone Superior, Thompson, Victoria, Sable, Lival, Alphonse Lavallée, Black Magic	350	refl	S	flav, anth, TSS,	VIP, regression coefficient (PLS)	berry	PLS (full bands), MLR (selected bands)	predict TSS, anth, total flavonoid	MLR: (flav, anth, TSS, selected, β-coefficient) R^2^ = 0.93, 0.97, 0.97; 0.93, 0.98, 0.86 VIP-PLS: R^2^ = 0.95, 0.99, 0.94	[83]
Touriga Franca, Tint-a Barroca, Touriga Nacional	2665	refl	S	sugar	No	berry	RR, NN, PLSR, 1DCNN	predict sugar content	R^2^ = 0.94 (1DCNN)	[84]
Touriga Franca (2012 and 2013)	324	refl	S	sugar	No	bunch	PLSR, NN	predict sugar content in new vintages	R^2^ = 0.93,0.92 (PLSR, NN, 2012); 0.95, 0.92 (PLSR, NN, 2013) for external	[85]
Touriga Franca (2012 and 2013)	324	refl	S	sugar	No	bunch	NN	predict sugar content (satisfactory generalization)	R^2^ = 0.906, RSME = 1.165 (2012); 0.959, RSME = 1.026 (2013)	[86]
Touriga Franca	240	refl	S	sugar, PH, anth	No	berry	NN	predict maturity parameters	R^2^ = 0.73 (PH), 0.92 (sugar), 0.95 (anth)	[87]
Touriga franca (TF, 2012 + 2013); Touriga nacional (TN, 2013); Tinta barroca (TB, 2013)	465	refl	S	PH, anth	No	berry	NN	predict PH and anthocyanin for new vintages and varieties	R^2^ = 0.72 (2013, TF, PH), 0.90 (2013.TF, anthocyanin)	[88]
Zalema, Syrah, Tempranillo	213	refl	S	TP, TA, sugar, PH	No	skin	MPLSR	screen and control maturity parameters	RSQ = 0.89 (TP), 0.99 (sugar), 0.98 (TA), 0.94(PH)	[89]
Globe grapes	360	drefl	SI	SSC	CARS, S-PA, UVE, GA, CA-RS-SPA, UVE-SPA	berry	PLSR	predict SCC	R2 c = 0.9775, R2 P = 0.9762	[90]
4 white and 3 red/black varieties	140	refl	SI	PH, TA, SSC	No	berry	PLSR	predict physical-chemical content and sensory	R^2^ = 0.95, 0.82 (TA); 0.94, 0.93 (SSC); 0.80, 0.90 (PH) for white and red/black grape	[91]
Kyoho grape	240	refl	S	DI, PH	SAE, SPA, CARS	berry	LSSVM, PLS	predict DI and PH	R^2^ = 0.923 (SAE-LSSVM)	[92]

Variety refers to grape varieties. No means the number of the sample in the research. Object means the object-wise: Single berry, a bunch, grape skin and grape seed. S/I means spectra and image features, respectively. Mod means the signal mode: Transmittance mode (trans), interactance mode (inter), reflectance mode (refl), diffuse reflectance (drefl), and diffuse transmittance (dtrans). Ext means the approach to selecting effective wavelength to build mode. Best result means the best performing model in the research. Application means the main relevant research contents in this cited literature. Reference means the reference resources.

##### Maturity Identification

Maturity estimation is critical for determining the grapes’ optimal harvest timing and storage mechanism. Study results have verified the possibility of maturity detection. Based on the spectral feature, Costa et al. used Linear discriminant analysis (LDA), Quadratic discriminant analysis (QDA), LDA_Mahalanobis and PLS-DA classification models to identify the three maturation stages of grapes (green, véraison, and ripe). PLS-DA performed best, with an accuracy of 93.15% [53]. Ribera-Fonseca et al. divided four different maturity clusters according to an Index of Absorbance Difference (I_AD_) of visible and near-infrared (VIS/NIR) spectroscopy (Cherry-Meter) by PCA, increasing level of maturity defined using technological parameters and anthocyanins concentration [55]. Virginia et al. collected white and red grapes in six stages according to the time. They divided them into three groups according to reducing-sugar content: Insufficiently ripe (1), optimally ripe (2 and 6), and overripe (3, 4 and 5) for white grape, and three groups: Young red wines (1), vintage reds (2, 5 and 6) and even sweet reds (3 and 4), respectively. PLS-DA was built for classification, and the percentage of correctly classified samples by the group was greater than 83% in all groups, except for the third stage in red grapes (79%) [56]. Baca-Bocanegra et al. used the NIRS technique combined with LDA, Discriminant partial least square (DPLS), and Pearson’s similarity index to predict the level of phenolic, flavanolic and anthocyanic compounds (divided into two classes by the extractable pyhsical-chemistry content). However, the study shows unremarkable results except for internal validation in 2017 season (especially for LDA, with an average accuracy of over 87%) [52]. Xiao et al. used VIS/NIR spectroscopy combined with SVM-DA based on the full and effective wavelengths (CARS and SPA) to discriminate five stages (green, pre-veraison, veraison, post-veraison, ripe). The SSC-based CARS-SVM-DA model showed classification accuracies of 90% and 100% for ‘Manicure Finger’ and ‘Ugni Blanc’ [54].

##### Seeded and Seedless and Geographical Origin Identification

Seedless grapes are essential factors affecting consumer preferences. The usual way is to tear the grapes to check the internal conditions of the grapes, which are time-consuming and laborious and cannot be used in large-scale industries. The spectral difference in the interaction mode can reflect the internal composition of the fruit. Based on the spectral feature, Kanchanomai et al. used the NIRS technique combined with the PLS-DA model to distinguish seeded and seedless grapes in the laboratory and field, with the best accuracy of 93.10% in the laboratory [49].

Attention to high-quality agro-products with a clear geographical origin is raising. Identity preservation is significant for the wine industry and market sector owing to many geographical classifications. There is a growing demand for analytical methods for tracing grapes and wines. Ferrer-Gallego et al. used NIRS combined with DPLS to distinguish the vineyard of origin using entire grapes, skin and seeds. The phenolic composition in grape skins and the grape seed was measured by HPLC-DAD/MS, and that of grape skins was regarded as phenolic in intact grapes. The model built by the seed obtained the best result, with the accuracy of 95%, and the excellent result was presented with entire grapes, with the accuracy of 93% [50].

##### Safety Inspection

Food safety has always been a concern for consumers. The food safety factors discussed here involve phytosanitary status evaluation and pesticide residues. There is an urgent need to develop non-contact techniques such as NIRS and HSI for food safety inspection. Based on the spectral feature, Beghi et al. used NIRS combined with PLS-DA to identify the phytosanitary status (healthy, sunburn, botrytis cinerea, powdery mildew, and sour rot), with the accuracy of 89.80%–94.00% [58]. Mohite et al. used HSI to detect grape of the no, single and double does pesticide residue. PCA, LASSO and Elastic Net were used to extract features, and ANN, SVM, RF and XGBoost were applied as the classification model. LASSO-SVM showed the best result, with the accuracy of 91.98% [59]. Ye et al. used VIS/NIR and NIR hyperspectral imaging to detect three grapes’ pesticide residues. SVM, LR, RF and Residual Network (ResNet) were used as predictive models. An outstanding performance was obtained, and the best result was over the accuracy of 90% [60].

#### 3.1.2. Quantitative Analysis

##### Quality Assessment

Based on the spectral feature of NIRS, many researchers have measured the physico-chemical composition to evaluate the quality and determine the optimal harvest time of the grape. Costa et al. used VIS/NIR reflectance spectroscopy to predict the quality and maturation attributes (TSS, total anthocyanins and yellow flavonoids) in wine grapes. Several pre-treatments were used, and PCR, PLSR and MLR were utilized as predictive models, which showed promising results for TSS and anthocyanins (R^2^ ≥ 0.90), and flavonoid content (R^2^ ≥ 0.70) [53]. Urraca et al. used NIR spectroscopy combined with PLSR under field conditions to estimate the TSS in grape berries. PCA was applied to improve outlier detection based on the 95% confidence ellipse. We used 1600 samples in the laboratory to explore the influence of the number of samples for the model. The result of calibration models built under laboratory conditions indicated that at least 700 berry samples are required to ensure enough prediction accuracy. Under field conditions, the prediction errors (RMSEP = 1.68°Brix, and SEP = 1.67°Brix) were close to those obtained with the laboratory dataset (RMSEP = 1.42°Brix, SEP = 1.40°Brix) [64]. Baca-Bocanegra et al. used a portable micro NIR spectroscopy combined with MPLS to screen extractable polyphenols (total phenolic, anthocyanins and flavanols) in red grape skin. However, the study showed poor results [52]. Xiao et al. used NIRS by comparison of benchtop Fourier-Transform (FT) and portable grating scanning spectrometers to predict SSC, with the best R^2^ of 0.918 in the region of 833–2500 nm [68]. Fernández-Novale et al. used on-the-go visible-short wave near-infrared (VIS+SW-NIR) spectroscopy combined with PLSR to monitor the composition (TSS, anthocyanin and total polyphenols), with the R^2^ of 0.95, 0.79 and 0.43 [66].

Besides, spectra data from HSI was used widely for quality assessment in grapes. For the prediction of anthocyanin, Fernandes et al. [74] and Gutiérrez et al. [78] detected anthocyanin content using hyperspectral imaging in the range of 400–1000 nm with PLSR and SVM, respectively, with SVM achieving a higher R^2^ of 0.83 (0.65 for PLSR). José et al. [33], Chen et al. [75] and Nogales-Bueno et al. [77] detected anthocyanin content using hyperspectral imaging in the range of 900–1700 nm, and research involved the Modified partial least squares regression (MPLSR), PLSR and Support Vector Regression (SVR), with the best R^2^ result of 0.94 when using SVR. For SSC, Benelli et al. [79], Xu et al. [80] and Benelli et al. [81] used HSI in the range of 400–1000 nm to detect the SSC content. PLSR, LSSVM and PLSR, and PLSR were used in three studies, respectively. Three studies obtained excellent results, and the best was the VMD-RC-LSSVM model, with R^2^ of 0.93 [80]. María et al. used on-the-go HSI in the vineyard to monitor grapes during ripening. TSS, TA, PH, MA, tartaric acid, anthocyanins, and total polyphenols were measured. PLSR was built to predict those parameters, with the $R_{P}^{2}$ (external validation) of 0.82 for TSS, 0.81 for TA, 0.61 for pH, 0.62 for tartaric acid, 0.84 for MA, 0.88 for anthocyanins and 0.55 for total polyphenols [82]. Gabrielli et al. used HSI to predict sugar, total flavonoid, and total anthocyanin contents. PLS with full wavelengths and MLR with optimal wavelengths selected the regression coefficients and VIP score based on PLS were built to predict the grape quality. RMSP based on PLS was 0.9, and this based on MLR with an optimal wavelength of regression coefficients reduced to 0.7°Brix [83]. Nogales-Bueno et al. used near infrared HSI combined with MPLS to predict and evaluate TP, TA, PH and sugar. PCA was applied to a preliminary inquiry for the latent structure of spectral matrix, all parameters obtained the great R^2^ result (>0.89) [89]. Besides, Rodríguez-Pulido et al. used HSI to determine flavanols in grape seed, and PLSR provide an R^2^ of 0.73 for total flavanols concentration, and 0.85 for predicting flavanols extracted with the model solution, and 0.88 considering a cultivar [45].

In addition to the above applications of traditional machine learning (ML) methods, deep learning (DL) is also used as a regression analysis model for quality evaluation. Based on the neural networks (NN) or 1DNN, several studies used HSI in the range of 380–1028 nm to detect the sugar content in grapes with different vintages and vineyards, and satisfactory results were presented, with an R^2^ of over 0.90% [84,85,86,87]. In Gomes et al. [85] and Gomes et al. [86], models were tested from the sample data in 2013, and satisfactory generalization was presented. In Gomes et al. [84], the external validation was developed by an independent test set. Gomes et al. used HSI combined with NN in estimating PH and anthocyanin content for new vintage and varieties and evaluated the generalization ability of HSI. Touriga Franca (TF), Touriga Nacional (TN) and Tinta Barroca (TB) were harvested in 2013, but only TF was collected in 2012. NN was built, trained and validated to predict PH and anthocyanin from the 2012 sample, and TF, TN and TB from 2013 were tested to evaluate generalization ability. The best R^2^ for PH was 0.72 for TF, and that for anthocyanin was 0.90 for TF. The study showed that if models are trained and generalized well, they would successfully be applied in new vintages and varieties [88].

##### Parameters Sensory Prediction

Basile et al. used Fourier-transform (FT) NIR spectroscopy to predict the main maturity parameters (SST and acid) and understand consumer preference driving factors in the “Victoria” and “Autumn Royal” grapes. The result showed sugars and acid content in “Victoria” were related to the appreciation. For “Autumn Royal”, there is no strong correlation [70]. Daniels et al. used FT-NIR combined with PLSR to detect maturity and sensory parameters (TSS, TA, TSS/TA, PH) and BrimA (TSS-K X TA) in different vintages and varieties. The optimal preprocess method was decided by the result obtained by the PLSR using 2016 data as the training set and 2017 as the testing set. The model obtained the R^2^ result of 0.71, 0.33, 0.57, 0.28, and 0.77 for TSS, TA, TSS/TA, pH, and BrimA, respectively [71]. Parpinello et al. used NIR spectroscopy to predict SSC and obtain information about consumer preference in ‘Italia’ table grape. PLSR was established and presented an acceptable result, with R^2^ of 0.85 for cross-validation and 0.82 for external validation. Discriminant Analysis (DA) was conducted to identify the class of preference by the NIR data, and the model performed a great result, with the accuracy of 78.5%, 98.7% and 75% for class 1, class 2 and class 3 [69]. The results showed that sensory prediction is a complex problem. It was mainly related to sugars, acids content, minor components and other “not flavoring” properties like color and texture, such as crunchiness, gumminess, etc., which is consistent with the literature [93].

#### 3.1.3. Conclusions

Regarding the mode of data acquisition, the discussed studies on NIRS include interactance, reflectance, diffuse reflectance and diffuse transmittance. Regarding HSI, the studies discussed adopted the reflectance. In terms of data preprocessing, PCA, as an unsupervised pattern recognition technique, has been widely used to reduce the dimension of spectra data and select effective wavelengths by the scores of PCA loading, and it shows the spatial distribution of the sample and presents and removes outliers [48,64,66,77]. Besides, Normalization [69,70], SG [69], SNV [71], MSC [49,71] and derivative processing [70,71], etc., have been applied in spectral data preprocessing methods to remove noise. Iteratively retains informative variables (IRIV) [80], Monte Carlo cross-validation (MCCV) [44], LASSO [59] and CARS-SPA [44] have also been widely used to select effective wavelengths to condense the data dimensions and to save computational time.

As for qualitative analysis, the results show a satisfactory performance for seeded and seedless and geographical origin identification, with an accuracy of over 93% [49]. For maturity identification, maturity stages were divided by TP, SSC and other maturity parameters. The classification method is most used by PLS-DA, and the results are acceptable. Besides, maturity identification is conducted using NIRS in those studies. Concerning the classification of grape and grape seed, the most widely used ML model is SVM, with the accuracy of over 81% [43], and the best is 99.3125% based on an EEMD-DWT denoising algorithm [44]. For safety inspection, the phytosanitary status and pesticide residues discussed here showed satisfactory results.

Concerning quantitative analysis, the above research has shown that the prediction result of TSS was better than that of PH, TA and other components [49]. For sensory evaluation, it is challenging considering the inadaptability for every determination of complicated internal and external attributes. For calibration models, PLSR [33,74,75,77,82,83], SVR [75,76,78], MLR [83] and Ridge regression (RR) are widely used. PLSR is the most popular chemometric method, followed by SVR and RR, with good results. However, PLSR does not always show the best results. In Xiao et al. [54], the results indicate that the LS-SVM model showed a better performance than PLSR. Besides, NN [87] and 1DNN [84] are widely applied, with strong generalization ability and stable results. The performance in the laboratory was better than the performance in the field [49,64]. This study presents the potential of using the HSI technique directly in the field through measurements under natural light conditions to predict the quality of grapes [64,81].

### 3.2. Image Feature Analysis

#### 3.2.1. Qualitative Analysis

For qualitative analysis, research based on image features for grapes is rare here. Grape grading is a vital sector in commercial production, and fine grading and exquisite packaging could effectively improve the value of goods. Image features are often used in qualitative analysis, such as identifying grape grades. Based on the image feature, Xiao et al. measured values of SSC, TP and color space CIELAB. Three classes (Class I, Class II, Class III) were established only by the values of SSC and TP due to poor correlation between CIELAB and spectral data. PLS-DA was built using VIS/NIR Spectroscopy data to identify four different grades of grape, and the accuracy was 77%–94% [51]. This study result is consistent with the previous literature because it is difficult to classify and evaluate grapes only by image features.

#### 3.2.2. Quantitative Analysis

For quantitative analysis, most researchers also used spectra information to assess grape quality, but image features were also applied to prediction. Firmness is the primary textural attribute of fruit and is commonly used to predict the quality of the grape. Based on the NIRS technology, Kanchanomai et al. used NIRS combined with PLSR to predict firmness, and different preprocessing methods were used for comparison. The best model the Savitzky-Golay first derivative (SGD1) for firmness, with the R^2^ result of 0.7427 and 0.7804 in the laboratory and field, respectively [49]. Xiao et al. used VIR/NIR spectroscopy to predict the L*, a*, b*, SSC and TP, and LS-SVM performed best, and all results were satisfactory [54]. Ribera-Fonseca et al. used a portable VIS/NIR spectroscopy device combined with Pearson correlation to evaluate firmness, and the R^2^ result of 0.89 was obtained [55]. Beghi et al. used a VIS/NIR spectroscopy combined with PLSR to monitor grape withering by measuring firmness, TSS and weight loss. The R^2^ and RPD results of 0.56 and 1.79 were obtained for firmness [73]. Color is a combination of visible light reflected or emitted from an object, influencing consumer preference. Xiao et al. used visible-near spectroscopy to grade for Manicure Finger and Ugni Blanc, and color space CIELAB was explored [51]. Besides, sensory parameters were explored. Raúl et al. used NIRS to evaluate sensory parameters, including firmness and color, and the study showed considerable potential for predicting the above sensory attributes [72]. Besides, based on the HSI, Baiano et al. applied HSI to predict the physico-chemical indices (seven grape varieties) and sensory. Sensory data was evaluated by a series of professional panels. To compare a hyperspectral image and RGB image, PLSR was also built using a combination of only red, green, and blue wavelengths and full wavelengths. For spectra data, the average R^2^ of all results was over 0.80. However, considering the selected three wavelengths, the correlation between sensory data and spectra information was poor. The research showed that HSI could predict physico-chemical indices, while it could not predict sensory data [91]. Min et al. used visible near-infrared HSI with deep learning, based on a regression approach to predict the PH and firmness. Stacked auto-encoders (SAE) was conducted to extract deep spectral features combined with pixel-level and mean spectra. PLS and LSSVM were established to predict firmness and PH. The best model is SAE-LSSVM, with the R^2^ p of 0.923. The study showed that SAE could be an alternative for dimensionality reduction and could predict the grape quality combined with HSI technology [92].

#### 3.2.3. Conclusions

Image feature has not drawn much interest in quality assessment compared to spectral feature. Regarding data preprocessing, in addition to the methods mentioned in Section 3.1, detrend was used [72], and SGD1 performed the best in Kanchanomai et al. [49]. Apart from the use in Section 3.1.3, PCA was conducted to research the latent structure of spectra data to identify the differences between the sample, and was behind the architecture of many food analysis devices through the analysis of reduced data [54]. In those studies, SPA [54,92], CARS [54,92], and VMD-RC [80] have also been applied to select characteristic wavelength and dimension reduction. Stacked auto-encoders (SAE) [92] has been used to extract the sample’s deep spectral features and obtained a satisfactory result. Similarly, for the calibration model, traditional ML methods, including PLS-DA [51], PLSR [49], and Pearson correlation [55], are used to analyze data and compare the performance between image features and spectral features. The comparison indicates the difficulty of only evaluating internally by image features due to the poor ability to characterize features about the internal information of fruit, while the corresponding spectral data model shows better results.

### 3.3. Fusion Data Analysis

#### 3.3.1. Qualitative Analysis

Data fusion strategies are performed as low-level, mid-level and high-level data fusion [94]. Low-level fusion is a direct concatenation of raw data from different sources. Mid-level fusion is a splice for features extracted from raw data. High-level fusion is the concatenation of the results from the built model [95]. Based on HSI, data fusion strategies have been used in the field. Here, we mainly discuss the fusion of a similar spectrum or the fusion of a map and spectrum.

Regarding qualitative analysis, the combination of spectral features and image features has been explored. For variety identification, Nogales-Bueno used anthocyanin profile, color image analysis, near-infrared hyperspectral imaging and data fusion (a low-level fusion of spectra and color space) to discriminate four autochthonous red grape cultivars. Stepwise-LDA was built as a classification model, with an accuracy of 88%, 54%, 100% and 100% for internal validation and 86%, 52%, 86% and 86% for external validation for four approaches, respectively. Image features performed poor results, and spectra and fusion data showed satisfactory results [57]. Data fusion was applied in the study, while the result is not better, and it is at least a comprehensive study.

#### 3.3.2. Quantitative Analysis

Concerning quantitative analysis, In Gao [90], GA, CARS, SPA, UVE and CARS-SPA, UVE-SPA were used to reduce dimensionality and extract the optimal wavelengths. The Grey-level co-occurrence matrix (GLCM) was applied to extract the texture feature and integrated it with image information (R, G, B, H, S, V, L, a, b). The result showed that SPA-PLSR combined with the fusion of image and spectra information (mid-level fusion) had the best detection, with the R^2^ of 0.9775 and 0.9762 for the calibration and prediction set [90]. In that study, the PLSR model using HSI technology obtained good results in the SSC determination of grape with the highest R^2^ P of 0.9763 based on fusion information in red globe grape. Besides, Orlandi et al. used low-level and mid-level data fusion strategies to condense the information obtained by an electronic eye (EE) and an electronic tongue (ET) sensing system for assessing grape ripening, and PLSR was used. The results showed the models using mid-level fusion performed better than that using EE and ET separately [96]. The studies here show that fusion data perform better than singe feature.

#### 3.3.3. Conclusions

The studies of data fusion are researched as a supplement and comparison to image and spectral features. The spectra and image features of the sample were acquired, while most of the studies only used spectral features. The studies have shown that only the RGB calibration model performance is limited [90,91]. Fusion data showed great feasibility in quality assessment and safety detection due to the acquisition of more features, which increases the accuracy of prediction for quality assessment [90]. A little research presented that the performance of fusion data is close to that of single spectral data, but it is also a comprehensive study [57]. Besides, color space, GLCM and spectral data are fused with various levels, and the multi-source information fusion of EE and ET showed great performance. For calibration models, traditional ML (PLSR and LDA) approaches were used for data analysis, and satisfactory results were obtained. Among them, the low-level and mid-level are used more, and the high-level fusion is the least researched for critical information loss due to the application of the model possibly. Besides, mid-level fusion has a better performance than low-level. Overall, spectra better characterize the features related to grape quality evaluation, while image features can only characterize visible features, such as color, damage, mildew, pests, etc. Thus, spectral features are more suitable and applicable to developing real-world applications than image features. Data fusion is a supplement for spectral data and has shown great potential in non-destructive detection [97,98,99,100,101,102].

## 4. Challenges and Prospects

In recent years, NIRS and HSI has been proven to be an effective tool due to its characteristics of fast, high accuracy and non-destructive compared with the traditional quality detection methods in agricultural and food products (AFP). The application of NIRS and HSI allows reliable and convenient monitoring of grape composition to facilitate the decision-making process dealing with grape quality sorting and harvest scheduling. While most maturity identification was conducted using NIRS, HSI can also be used for maturity identification due to the catch of color and component changes during ripening. HSI can provide spatial attributes of agricultural produce for visual examination and further image analyses, and a combination of HSI and detection of internal sugar, moisture, surface pesticide residues, etc., was researched by many researchers and obtained satisfactory results. Besides, the visualization prediction technology of HSI provides excellent convenience for actual industries and applications.

The current hyperspectral imaging technology mainly faces significant challenges. Regarding the data processing, hyperspectral imaging data is high volume and redundant, which usually requires various effective algorithms to extract feature wavelengths for dimensionality reduction, making data preprocessing for noise reduction, and robust calibration models for extracting deep features. As summarized above, the preprocessing and wavelength extraction methods are determined by actual performance. In general, SG, SNV and MSC are common and efficient pre-treatment methods, and PCA and SPA are used widely for wavelength extraction and perform acceptable results. Besides, Deep learning (DL) shows the remarkable ability of feature extraction. However, the spatial information obtained through hyperspectral imaging is not fully utilized, while it contains some critical information. The data fusion technology could extract more comprehensive information, including internal hydrogen-containing group information (e.g., O-H, C-H, and N-H) and external image information, and it always has a good performance [57,90,91,100].

Robust calibration models have always been the focus of researchers. Various ML models have always obtained excellent and acceptable results. However, there is little research on generalization ability. DL have performed excellent quality inspection results and good generalization ability due to the remarkable advantage of processing high-throughput data rapidly and extracting sample features automatically [86,88]. In particular, one-dimensional convolutional neural networks (1D-CNN) are widely used in agro-product quality, and excellent results have been obtained [103,104,105,106]. Besides, the deep convolutional generative adversarial network (DCGAN) was applied in calibration models due to limited or unbalanced data sets, which effectively increased the size and variability of the sample [107]. The low robustness affects the development from laboratory to field application, one variety to another variety, one vintage to another vintage and one device to another device, etc. Many variations exist seriously in nature, and it is complex and inevitable. Thus, we could build various sample databases from different vintage, vineyards, and varieties to meet more variations.

There are many machine learning algorithms used in relevant research, and different methods have shown different effects. For spectral and hyperspectral data from different sources, the data is very complex and diverse. The problem we are facing now is how to deal with these complex problems using existing and new data analysis methods in practical application of grape quality detection. In addition, at the same time, new and various algorithms have developed rapidly. For example, DL is used to effectively extract data features and establish better models [108]; transfer learning can achieve model migration in different application scenarios (different varieties, years and instruments, etc.) [109]; and Automated Machine Learning (AutoML) can be developed to achieve the automation of ML to free up developer’s time to focus on other tasks [110], etc. With the help of these methods, we aim to develop a more robust and universal model, and it might be a future research trend. Besides, the hyperspectral imaging system is currently expensive and ponderous, which is not conducive to promotion and application. The main working range is still near the ground. It is difficult to establish interconnection with Unmanned Aerial Vehicles (UAV) and satellite regional remote sensing to realize large-scale collection and detection of outdoor trees and fruits. This restricts the application of the HSI system in real-time industrial applications. It is necessary to customize the equipment by selecting characteristic bands and developing cheaper equipment.

Overall, as an effective non-destructive testing technology, hyperspectral imaging will still play a significant role in the agro-food industry as a critical research tool. Further development and applications will follow that help the agricultural industry meet food safety and quality inspection needs. The main strategies in the future were proposed to develop rapid, high-precision, real-time and low-cost detection systems for the food industry, and it will be the problem we will continue to work on for a long time in the future. The specific potential and results need to continue to be explored in future research.

## 5. Conclusions

This review stressed the recent progress of NIRS and HSI in identifying variety, vintage, and geographical origin, and assessing quality attributes, biochemical components and sensory parameters of table grapes. Qualitative and quantitative analysis is compared and summarized regarding signal mode, data preprocessing method, calibration model and result performance. Spectral information, image information and fusion data can be explored based on ML and DL to link the measured reference values, and optimal prediction models could be selected to quantify the enological parameters. The research has shown that NIRS and HSI combined ML have great potential in the quality detection of grapes, which is helpful to fully utilize grapes of all grades and benefits sustainable economic development. The remarkable advantage of this technique is chemical-free and non-destructive detection, together with the abundant information provided, which enables multiple quality features to be examined. In the future, with continued technical innovations in manufacturing and computing, NIRS and HSI performing in a low-cost and high-speed way for online and real-time detection of various products foreseen. More work must be done to implement this technology to achieve real-time applications successfully.

## Figures and Tables

**Table 1:** Summary of the difference and connection between the NIRS and HSI systems.

Tech ^1^	Difference		Connection	
	Instrument	Data	Application	Data Process
NIRS	Lower cost; portables	Spectra	Evaluate chemical parameters; on-lining inspection,	Rely on ML ^2^, chemo-metric model
HSI	Higher cost; ponderous	Spectra and image	Evaluate chemical and physical parameters; visualize map,	Poor robustness and adaptability; difficulty in valid information mining

^1^ Tech means NIRS and HSI techniques. ^2^ ML means machine learning.

## Data Availability

Not applicable.

## Abbreviations

1D-CNN	One-dimensional convolutional neural networks
ANN/NN	Artificial neural networks
Anth	Total anthocyanins
CARS	Competitive adaptive reweighted sample
DI	Durofel index (berry firmness)
DL	Deep learning
DM	Dry matter
DPLS	Discriminant partial least square
DWT	Discrete wavelet transform
EEMD	Ensemble empirical mode decomposition
Flav	Flavanols
GLCM	Grey-level co-occurrence matrix
GDA	General discriminant analysis
IRIV	Iteratively retains informative variables
LAB	color space values
LDA	Linear discriminant analysis
LV	Latent variable/factors
MA	Malic acid contents
MCCV	Monte Carlo cross-validation
ML	Machine learning
MPLSR	Modified partial least squares regression
MSC	Multivariate scattering correction
PCA	Principal component analysis
Pearson	Pearson’s similarity index (1/(1 − R^2^))
PLS	Partial least squares analysis
PLS-DA	PLS discriminant analysis
PLSR	Partial least-square regression
QDA	Quadratic discriminant analysis
RF	Random forest
ResNet	Residual Network
RR	Ridge regression
RSQ/R^2^	Coefficient of determination
SAE	Stacked auto-encoders
SG	Savitzky-Golay smoothing
SNV	Standard normal variate transform
SSC	Soluble solids content
SVR	Support vector regression
TA	Titratable acidity
TB	Tinta Barroca
TF	Touriga Franca
TN	Touriga Nacional
TP	Total phenolic
TS	Total sugars
TSS	Total soluble solids
VIP	Variable importance in projection
VMD-RC	Variational mode decomposition (VMD)-regression coefficients
